# Effect of dietary fiber content on nutrient digestibility and fecal microbiota composition in growing-finishing pigs

**DOI:** 10.1371/journal.pone.0206159

**Published:** 2018-10-24

**Authors:** Mathilde Le Sciellour, Etienne Labussière, Olivier Zemb, David Renaudeau

**Affiliations:** 1 PEGASE, INRA, Agrocampus-Ouest, Rennes, France; 2 GenPhySE, Université de Toulouse, INRA, INPT, INP-ENVT, Castanet Tolosan, France; University of Illinois, UNITED STATES

## Abstract

Microbial population in the gastrointestinal tract plays a central role in health and nutrient digestion. The objective of the present study was to investigate the relationships between microbiota and apparent digestibility coefficients with respect to age and diet. Pigs from Large-White, Duroc or Pietrain breeds were raised under the same housing conditions and fed alternately a low-fiber (LF) and a high-fiber diet (HF) during 4 successive 3-week periods. Data collection for digestibility measurements was achieved during the last week of each period. At the end of each period, fecal microbiota was collected for 16S rRNA gene sequencing. The microbiota remained stable across periods whereas digestibility of energy, crude proteins and cell wall components increased. The microbiota was resilient to diet effect and pigs fed the LF diet were discriminated to those fed the HF diet using 31 predicting OTUs with a mean classification error-rate of 3.9%. *Clostridiaceae* and *Turicibacter* were negatively correlated whereas *Lactobacillus* was positively correlated with protein and energy digestibility coefficients in the LF group. In addition, *Lachnospiraceae* and *Prevotella* were negatively correlated with cell wall component digestibility. In contrast, no significant correlation was found between microbiota composition and digestibility coefficients when pigs were fed the HF diet. Interestingly, it was also no longer possible to distinguish animals from different breeds once the animals were fed a HF diet, so that the microbiota could only trace the breed origin in the first period and in the LF group. In our experimental conditions, 3 weeks of adaptation to a new diet seems to be sufficient to observe resilience in growing pigs’ microbiota. We demonstrated that fecal microbiota can be used to classify pigs according to their dietary treatment. Some bacteria are favorable or unfavorable to digestibility. This suggests that manipulations of bacterial populations can improve digestibility and feed efficiency.

## Introduction

Microbial population in the gastrointestinal tract plays a central role in health and nutrient digestion of the host. A major function of gut microbiota is to break down indigestible carbohydrates into short-chain fatty acids (SCFAs), particularly acetate, propionate and butyrate. These SCFAs are an important energy source for the host. In growing pigs, energy produced from hindgut fermentation of dietary fiber represents 7 to 18% of the total available energy absorbed by the pig [1]. In addition, the positive effect of microbiota on gut health status [2] can be related to the physiological effects of SCFAs [3]. According to Cornick et al. [4], butyrate is a potent inhibitor of inflammation, tumor growth and also stimulates mucus production and secretion with subsequent positive effects on gut health.

The gut microbiota composition changes according to factors related to the animal such as age [5, 6], health status [7, 8] or growth performances even though 1% of the genes of the gut microbiota are shared between all the pigs [9]. According to Ramayo-Caldas et al. [10], microbial clusters dominated either by *Prevotella* or *Ruminococcus* genera allow to discriminate pigs with high or low average daily gain. Environmental factors can also lead to gut microbiota modifications through feeding management or sanitary status of the farm [11], in-feed antibiotics [12] or diet composition. Dietary fiber content seems to be a major driver of gut microbiota composition [13]. Numerous studies have already focused on the relationship between dietary fiber and pig gut microbiota [8, 14, 15]: addition of resistant starch increases total bacterial counts and stimulates healthy gut-associated butyrate-producing *Faecalibacterium prausnitzii* growth in the colon [15] even though the impact of repeated changes in fiber content in the pig diets has not been evaluated yet.

In growing pigs, the nutrient digestibility increases with the age [16], is affected by the breeds [17] and is mainly influenced by dietary management. The digestibility of energy linearly decreases by about 0.8% for each extra percentage of neutral detergent fiber dietary content [18–20]. According to these authors, this detrimental effect of dietary fiber is smaller in adult sows when compared to growing pigs. It appears that factors influencing the microbiota composition also affect nutrient digestibility. However, little is known regarding the subsequent consequences of changes in gut microbiota nutrient digestion and metabolism in swine. Data about pig gut microbiota and fiber digestibility remain scarce, but 11 bacterial genera were positively correlated with apparent crude fiber digestibility in Chinese pigs fed low dietary fiber [21].

The objectives of the present study were to investigate the effects of dietary fiber content on gut microbiota composition and nutrient digestibility in pigs at different ages and to evaluate the relationships between digestibility and microbiota composition. Pigs originated from 3 different breeds and farms to obtain robust findings.

## Material and methods

This study was conducted in accordance with the French legislation on animal experimentation and ethics, and this research was authorized by the French Ministry of Agriculture to conduct experiments on living animals at the INRA facilities in Saint Gilles, France (agreement 2015091517437619).

### Animals and experimental design

**Fig 1:**
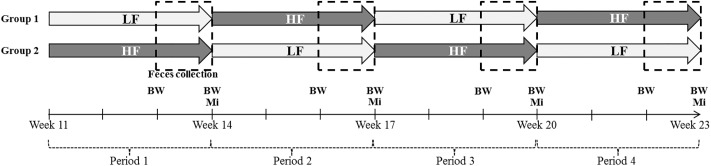
Experimental design and changes in the dietary treatments according to the experimental period. LF and HF are for low and high fiber diets, respectively. Each one-week feces collection period (dashed squared) was preceded by 2 weeks of adaptation to the experimental diet. Feces samples for microbiota (Mi) analysis were collected on the last day of collection period, and body weights (BW) were recorded at the beginning and at the end of the feces collection period.

A total of 22 Duroc, 20 Large White and 21 Pietrain castrated pigs were used in the study conducted in 2 successive replicates. Each breed originates from a different farm (Large-White pigs originated from the experimental facility of INRA, St Gilles, France. Pietrain pigs came from EARL Lucien Geffrelot, Lamballe, France. Duroc pigs came from EARL Maguer, Elliant, France) and the animals were transferred to our experimental facilities at 20 days of age. All three breeds were raised in our facilities under the same conditions until the start of the experimental period at 11 weeks of age and 29 kg, 35 kg and 37.2 kg mean live body weight (BW) for Duroc, Large White and Pietrain pigs, respectively. Total tract digestibility measurements were conducted over four consecutive 3-week periods (Fig 1). During each collection period, pigs were housed individually in digestibility cages for a 7-d collection of feces following 14-d of adaptation to the dietary treatment (collection started at 13, 16, 19 and 22 weeks of age). In the 10 first days of adaptation, pigs were housed individually in metal slatted cages (77 × 125 cm). Four days before the starting of the collection period pigs were moved to metabolic slatted cages (52 × 117 cm). The control low-fiber diet (“LF diet”) contained 2.8% of crude fiber and was formulated with cereals (corn, barley and wheat) and soybean meal. The increase in dietary fiber content in the high fiber diet (“HF diet”) was obtained by a partial substitution of corn and soybean meal by wheat bran, rapeseed meal, and soya hulls (Table 1). This substitution introduced different types of fibers in order to be close to the situations encountered in pig diet in commercial conditions. The ratio digestible lysine/net energy was kept at 0.86% for both diets; amino acids/lysine ratios were similar between diets as well as the digestible phosphorus and the calcium/digestible phosphorus ratio. Pigs received alternately LF and HF diets during the four 3-weeks periods, starting by LF diet for half of the animals (each breed equally represented) and HF diet for the others (Fig 1). During the adaptation and the collection periods, fresh diets were offered twice a day as pellets at feeding scale close to the *ad libitum* level. The negligible restriction allowed to homogenize feed intake between pigs within a same breed and to limit feed refusals and spillage that may generate subsequent errors in digestibility measurements. However, it slightly reduced the growth performance compared to the expected ones in pigs fed *ad libitum*. Feed allowance was constant over each 7 days of the collection period and increased between two collection periods in order to adjust to the feeding scale for spontaneous change in voluntary feed intake with BW. Pigs had free access to water. The space allowance in the cage was adjusted each period to optimize the comfort of the animal and the quality of the excreta total collection. Room temperature was set to 24°C.

**Table 1:** Composition of the experimental diets^1^.

	LF	HF
**Ingredients, %**		
Corn	34.58	17.77
Barley	17.77	17.77
Wheat	17.77	17.77
Soybean meal	15.74	9.18
Rapeseed meal	-	1.97
Wheat bran	2.50	15.00
Soybean hulls	-	10.00
Sugar beet pulp	-	5.00
Cane molasses	1.03	0.68
Corn starch	4.25	-
Sunflower oil	1.00	-
L-Lysine HCL	0.33	0.25
L-Threonine	0.15	0.10
L-Tryptophan	0.03	0.01
DL-Methionine	0.08	0.03
Sodium chloride	0.45	0.45
Calcium carbonate	0.82	0.62
Dicalcium phosphate	1.20	1.11
Minerals and vitamins	0.50	0.50
Indigestible markers	1.80	1.80
Digestible Lysine / Net energy	0.86	0.86
**Analyzed chemical composition, %** ^2^		
Ash	4.9	5.6
Crude protein	14.4	14.4
Crude fat	3.7	2.7
Crude fiber	2.8	7.7
Neutral detergent fiber	10	20
Acid detergent fiber	3.4	9.5
Acid detergent lignin	0.7	1.4
Gross energy (MJ/kg)	16	16

^1^ LF: low-fiber diet; HF: High-fiber diet.

^2^ Chemical compositions were adjusted for a dry matter content of 88%

### Measurements

Cumulated feed intake and cumulated potential refusals or spillage over each 7-d collection period were recorded, and the dry matter (DM) of proposed weekly feed and refusals were measured. At the end of each replicate, the 4 feed samples were pooled for further chemical analyses. The BW was measured at the beginning and the end of each collection period. Feces were daily and totally collected once a day, cumulated during the whole collection period and stored at 4°C [18, 22]. At the end of each collection period, feces were weighed, homogenized and three separate samples were prepared, one for freeze drying and further chemical analyses and the others for immediate DM determinations. At the end of the 7 d-collection period, fecal samples were collected aseptically from individual pigs in plastic bags. Fecal samples were thoroughly homogenized and 2 aliquots were frozen with liquid nitrogen until transferring them within 10 minutes to a freezer to be stored at -80°C until analyses.

### Chemical analysis and DNA sequencing

Pooled samples of feed and the individual fecal samples were analyzed for DM, ash (incineration at 550°C), nitrogen (N; Dumas method), Weende crude fiber (CF) and gross energy (IKA adiabatic calorimeter) using the methods of AOAC [23]. Cell wall fractions [Neutral Detergent Fiber (NDF), Acid Detergent Fiber (ADF) and Acid Detergent Lignin (ADL)] were determined according to the method of Van Soest et al. [24]. Feed samples was also characterized for their fat content using petroleum extraction with prior acid hydrolysis according to AOAC [23].

Microbial DNA was extracted using beat-beating with the Zymo kit from 50 mg of feces sample according to a method described previously [14]. The V3-V4 region of the 16S rRNA gene was amplified using F460 and R460 primers (F460: CTTTCCCTACACGACGCTCTTCCGATCTACGGRAGGCAGCAG, R460: GGAGTTCAGACGTGTGCTCTTCCGATCTTACCAGGGTATCTAATCCT) and 30 cycles of PCR with an annealing temperature of 65°C. Homemade 6-bases index was incorporated during a second PCR to perform multiplexing. Resulting libraries were pooled by 2 × 250 bp paired-end sequencing on Illumina MiSeq platform according to the manufacturer instructions. Sequences were then paired and cleaned internally in the GeT-PlaGE platform (Toulouse, France) for length, homopolymers and undetermined nucleotides. The quality of the sequences was controlled by adding 4 mock samples of known composition to the run. The quality of the run was checked internally using PhiX, and then each pair-end sequences were assigned to its sample with the help of the previously integrated index. Then proportion of the species was checked after assembly using the FLASH software with at least 10bp overlap and at most 10% mismatch.

Chimeras were removed from the sequences dataset before to be clustered de novo into Operational Taxonomic Units (OTUs) using VSEARCH [25] with a threshold of 0.97 similarity. A table of relative abundance of each OTU in each sample was created. Usearch9.2.64_i86linux32 was then used for taxonomic assignation based on SILVA database [26].

### Calculations and statistical analysis

Related to the pigs’ performance, the average daily BW gain (ADG) was calculated as the difference between the final and the initial BW of each collection period. The daily feed intake was calculated by the difference between feed allowance and feed refusal. The average daily feed intake (ADFI) was the average value from all the duration of collection period. Feed conversion ratio (FCR) was calculated as the ratio between ADFI and ADG and standardized for the dry matter content.

Hemicellulose and cellulose contents in feed and feces were calculated as the difference between NDF and ADF contents, and between ADF and ADL contents, respectively. Apparent digestibility coefficients of organic matter (OM), energy, N, hemicellulose, cellulose and lignin were calculated for each pig and each period [27].

Statistical analysis was run under R 3.3.2 except for ANOVA models for which SAS (version 9.4, SAS Inst. Inc., Cary, NC) was used. A summary of the pipeline analysis is shown in S1 Fig.

Performances traits and digestibility coefficients were analyzed using the mixed model of SAS including the effects of breed (n = 3), diet (n = 2), period (n = 4) and interactions. The effects of breed, diet and collection period were considered as fixed effects. The REPEATED statement was included in the model for accounting for within-pig covariability of repeated measurements made on the same experimental unit during the four consecutive collection periods. Differences between means were assessed through the statistical test of Tukey’s least significant difference.

Phyla abundance among diets, breeds and periods was analyzed through chi-squared tests. Diversity of the microbiota was described using the Simpson and the Shannon indexes (vegan R package) on the dataset previously rarefied to 5,000 sequences per sample. Samples under 5,000 sequences have been taken out of statistical analyses. The effects of diet, breed and period on these indexes were analyzed by ANOVA. The mixed model of SAS included the fixed effects of breed, diet and period. The REPEATED statement was included in the model to account for within-pig covariability of repeated measurements during the four consecutive collection periods.

The effects of the different factors on OTUs were assessed with generalized linear models (glm) using edgeR package in R. This method takes care of multiple factors by fitting glm with the design matrix of the experiment. The dispersion of the OTU abundance is estimated with accuracy, since all sources of variation are accounted for. For example, to extract differentially abundant OTUs between diets, the glm model took into account the variation related to the replicate, breed and period factors. The p-values were then adjusted with false discovery rate (FDR) method. The significance threshold of the adjusted p-value was 0.05. Unfortunately, GLM models from edgeR do not take into account the repeated measures.

The proportions of the explained variance by diet, breed, period or repetition factors are evaluated through the Bioconductor R pvca package.

A Sparse Partial Least Square Discriminant Analysis (sPLS-DA) from MixOmics R package was performed. This method enables to assess the minimal number of OTUs required to predict the group the animals belonged to according to their diet, breed and period. The multilevel parameter in sPLS-DA function is added to take into account the repeated measures for every animal. The OTU table was normalized through Total Sum Scaling (TSS) and Centered Log Ratio (CLR) transformation. Error-rate of the model and stability of the discriminant OTUs was evaluated by M-fold cross-validation in sPLS-DA analysis with 10 folds and repeated 10 times. The OTUs were considered stable if selected in more than 90% of the cross-validation tests.

Pearson correlations between energy, N, cellulose and hemicellulose digestibility coefficients and the OTUs were calculated and p-values were adjusted with FDR method for accounting for multiple tests. The correlation was considered significant when the associated p-value was under 0.05.

## Results

### Animal performance and digestibility coefficients

At the end of the experiment, data from 243 collection periods were available. Three animals died before the end of the trial and one collection period was missing for 4 pigs due to sickness. Data related to animal performance and digestibility coefficients are presented in Table 2. From the analysis of variance, the effect of the repetition was significant (P < 0.05) only for the mean BW. These data were not discussed and are only indicative.

**Table 2:** Effect of diet, breed and period on growing and feeding performances in growing pigs^1^.

	Diet		Breed			Period				RSD^2^	p-values^3^					
	LF	HF	DU	LW	PI	1	2	3	4		Diet	Breed	Period	DxB	DxP	BxP
n	123	120	80	79	84	64	59	60	60							
BW (kg)	54.5	54.8	50.1^a^	55.4^b^	58.2^c^	35.8^a^	48.6^b^	61.0^c^	74.3^d^	3.0	0.337	<0.001	<0.001	0.924	0.006	0.734
ADFI (g/d)	1414	1405	1367^a^	1465^b^	1397^c^	1242^a^	1371^b^	1462^c^	1573^d^	349	0.355	<0.001	<0.001	0.979	0.967	<0.001
ADG (g/d)	673	570	595	621	649	613^ab^	669^a^	560^b^	647^a^	136	<0.001	0.231	0.001	0.034	0.191	<0.001
FCR	2.56	3.18	2.94	2.94	2.73	2.41^a^	2.68^ab^	3.40^c^	3.00^bc^	1.06	<0.001	0.670	<0.001	0.709	0.312	0.025
**Digestibility coefficients (%)**																
OM	88.3	80.6	84.5	84.3	84.8	82.8^a^	84.1^b^	85.4^c^	85.9^c^	1.7	<0.001	0.211	<0.001	0.240	<0.001	0.061
Energy	86.5	78.3	82.4	82.2	82.7	80.7^a^	82.1^b^	83.3^c^	83.8^c^	1.8	<0.001	0.223	<0.001	0.336	<0.001	0.073
N	86.0	76.1	80.7^a^	80.8^ab^	81.8^b^	78.2^a^	80.9^b^	82.1^c^	83.4^d^	2.5	<0.001	0.014	<0.001	0.572	0.424	0.089
Cell wall																
NDF	56.5	60.3	58.4	57.6	59.1	52.8^a^	56.9^b^	61.1^c^	63.1^c^	5.3	<0.001	0.101	<0.001	0.172	0.049	0.002
Hemicellulose	58.8	60.1	59.5	58.9	60.0	54.2^a^	58.4^b^	62.4^c^	63.2^c^	4.7	0.002	0.206	<0.001	0.072	0.816	<0.001
Cellulose	50.9	63.2	57.1	56.0	57.8	50.7^a^	54.7^b^	59.5^c^	63.4^c^	7.8	<0.001	0.272	<0.001	0.094	0.002	0.029
Lignin	43.8	56.5	50.3	49.2	51.2	46.7^a^	50.7^bc^	50.2^b^	53.6^c^	7.0	<0.001	0.143	<0.001	0.104	0.022	0.095

^1^ LF and HF = low and high fiber diets, respectively. DU, LW, PI for Duroc, Large-White, and Pietrain breed, respectively. n = number of observations, BW = mean live body weight during the collection period, ADFI = average daily feed intake, ADG = average daily gain, OM = organic matter, N = nitrogen, NDF = neutral detergent fiber, hemicellulose: calculated as the difference between neutral detergent fiber and acid detergent fiber, cellulose: calculated as the difference between acid detergent fiber and acid detergent lignin, lignin: acid detergent lignin.

^2^ Residual Standard Deviation

^3^ From the ANOVA with diet effect (D, n = 2), breed effect (B, n = 3), period effect (P, n = 4), and interactions. Least square means within one column for the different breeds and periods with the same letter exponent do not differ (P < 0.05).

**Fig 2:**
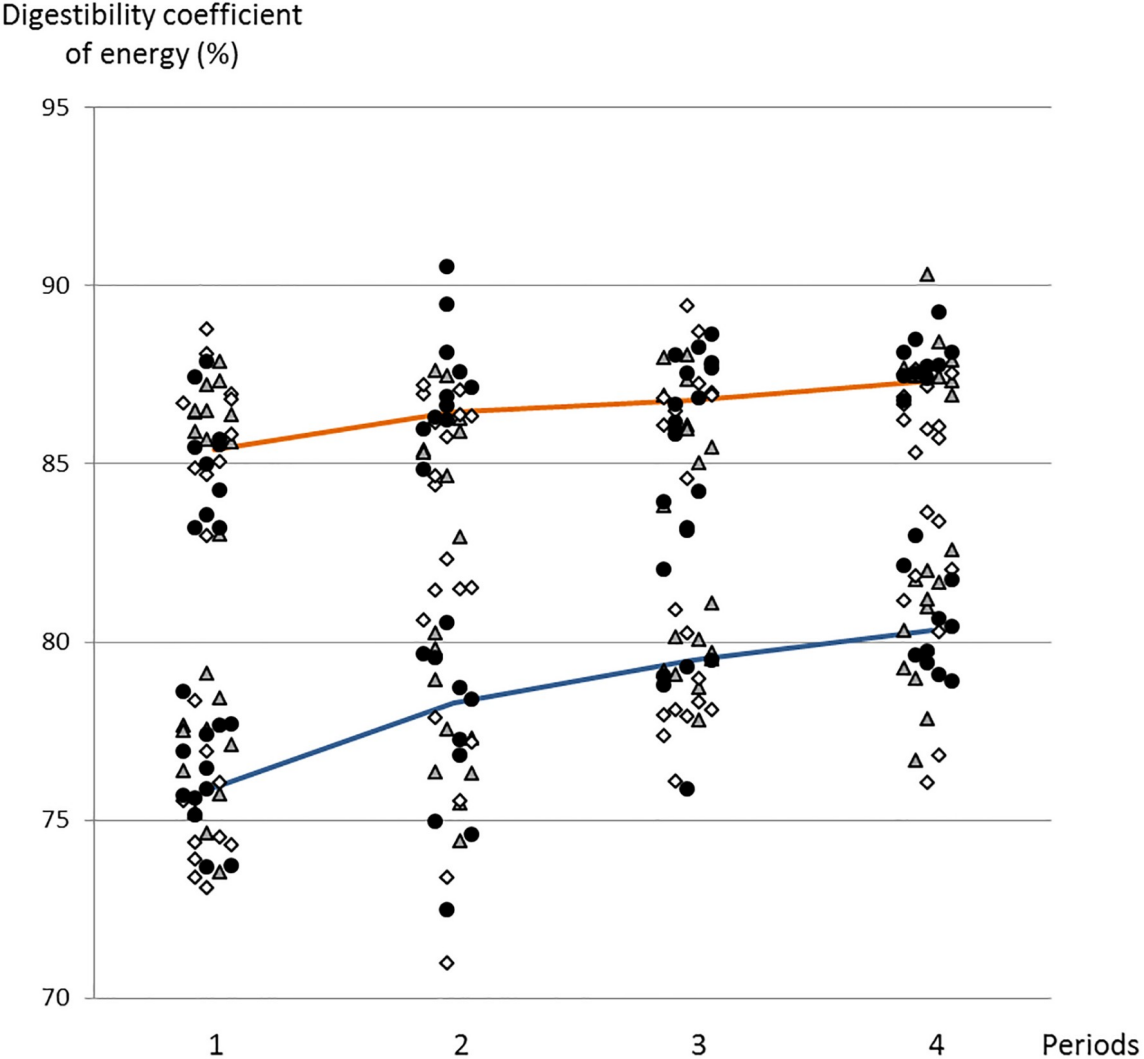
Evolution of digestibility coefficient of energy across periods 1 to 4 for the low-fiber (orange) or high-fiber (darkblue) diets in Duroc (▲), Large-White (◇) and Pietrain (●) pigs.

The digestibility increased linearly over time (Table 2 and Fig 2). From the first to the fourth collection periods the digestibility of OM, energy and N reached 3.7, 3.8 and 6.6% improvement, respectively, whereas NDF, hemicellulose, cellulose and lignin digestibility coefficients reached 19.5, 16.6, 25.0 and 14.8% improvement, respectively. In Pietrain and Large White breeds, the digestibility of NDF and hemicellulose significantly increased (P < 0.05) from the first to the third collection periods but remained stable thereafter. In contrast, Duroc pigs had stable digestibility coefficients during the first two collection periods and increased linearly during the third and the fourth periods. The digestibility of OM, energy, N, cellulose and lignin (ADL) tended to follow the same evolution as NDF and hemicellulose. This leaded to a significant interaction between breed and period for the digestibility of NDF, cellulose and hemicellulose (P < 0.05). No interaction between breed and diet has been observed (P > 0.07).

The digestibility of OM, energy, N and lignin were 5 to 10% higher in the LF diet (88.3%, 86.5%, 86.0% and 56.5% vs 80.6%, 78.3%, 76.1% and 43.8%, respectively) (Table 2). In contrast, the digestibility of NDF, cellulose and hemicellulose were 5 to 10% higher in the HF diet (60.3%, 60.1% and 63.2% vs 56.5%, 58.8% and 50.9%, respectively in LF diet).

### Microbial data

A total of 243 feces samples have been collected over the four periods. Among them 229 samples reached more than 5 000 sequences per sample and were kept for further analyses. Maximum number of sequences per sample was 46,827, with an average value of 18,934 ± 10,686. The sequences clustering generated 2,780 OTUs, essentially *Firmicutes* (68.3% of total sequences) and *Bacteroidetes* (23.4%) (S2 Fig).

Diversity indexes were independent from the period, the breed and the diet (S3 Fig). However, regarding the variance of the gut microbiota composition, 21%, 1.5% and 32% of the total variance was explained by the effects of the period, the breed and the diet, respectively.

We did not observe a constant shift in the microbiota composition over periods within each dietary treatments as shown by the absence of differentially expressed OTU over time based on the GLM analysis or our inability to discriminate microbial communities between periods (above 50% error-rate) (S4 Fig).

**Fig 3:**
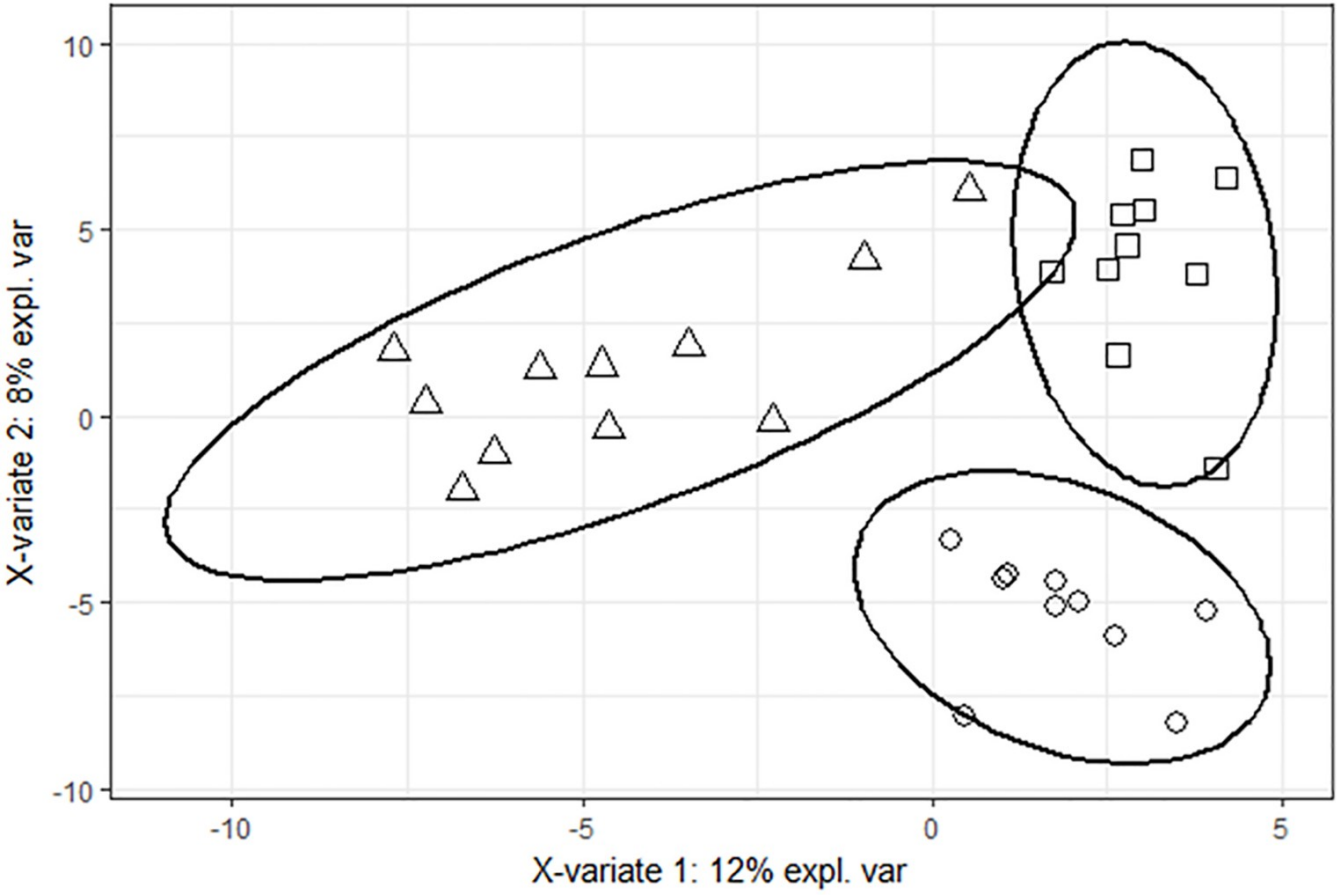
Score plot of two-component sPLS-DA model showing feces samples clustering according to the breed with percentage of variance captured for each principal component (Δ: Duroc, □: Large-White, O: Pietrain) for the animals fed low-fiber diet during period 1. According to the cross-validation permutation test, the misclassification error-rate was 14%.

**Fig 4:**
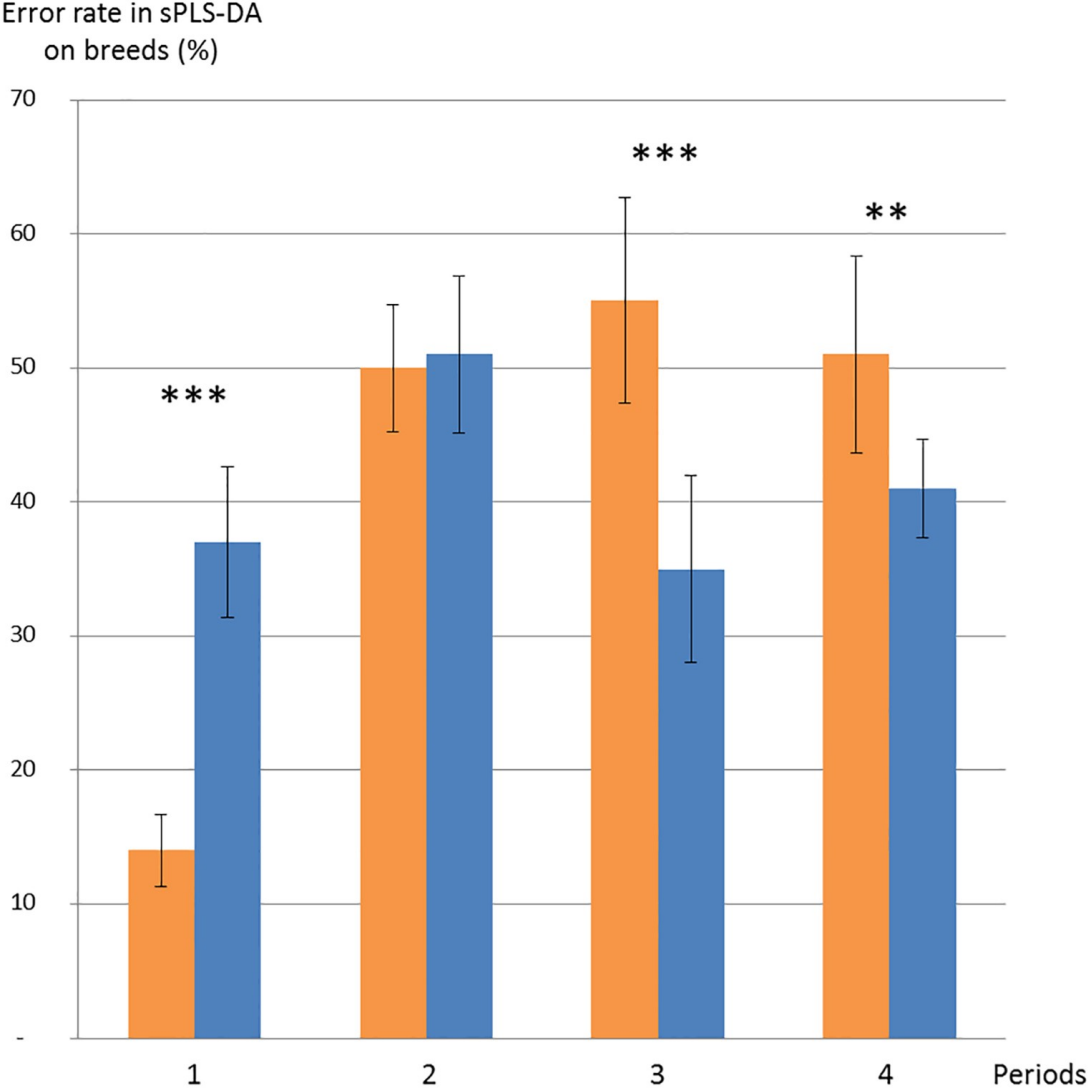
Discrimination of the breed through sPLS-DA across periods 1 to 4 for the low-fiber (orange) or high-fiber (darkblue) diets. T-test resulting in p-value < 0.01: ***; p-value < 0.001: ***.

Pigs fed the LF diet during the first collection period could be divided into three distinguished breeds by using the OTU abundance table (Fig 3). A total of 200 OTUs were selected to minimize the misclassification error-rate (i.e., 13.9%). This misclassification error-rate was based on the comparison, for each pig, between the actual breed and the predicted breed obtained from the sPLS-DA model. For the others collection periods, microbiota composition failed to identify separations between breeds (Fig 4), suggesting that exposure to the HF diet abolishes the difference between breeds.

**Fig 5:**
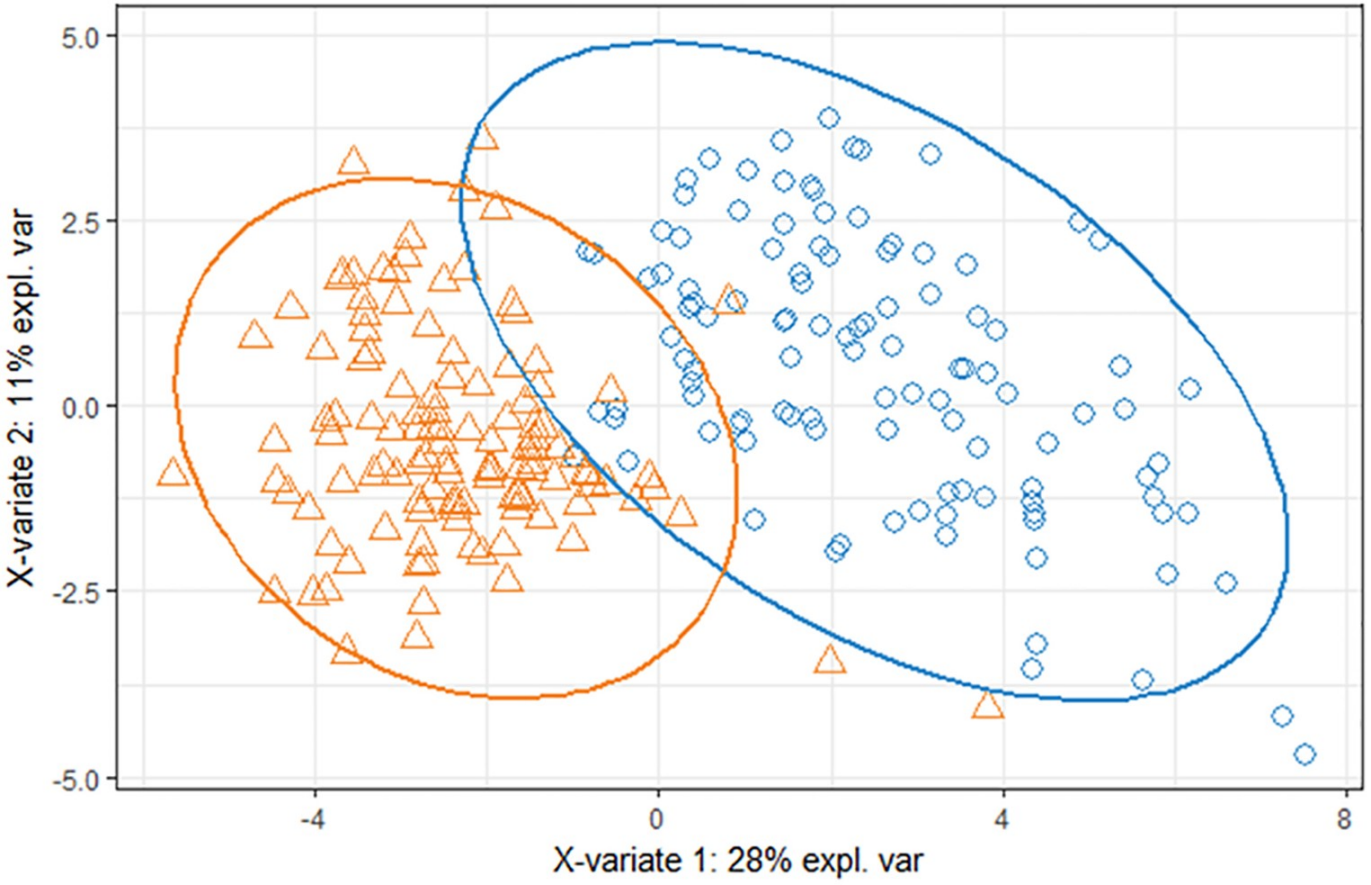
Score plot of two-component sPLS-DA model showing feces sample clustering according to the diet with percentage of variance captured for each principal component (Δ: Low-fiber diet, o: High-fiber diet). According to the cross-validation permutation test, the misclassification error rate was 3.9%.

**Fig 6:**
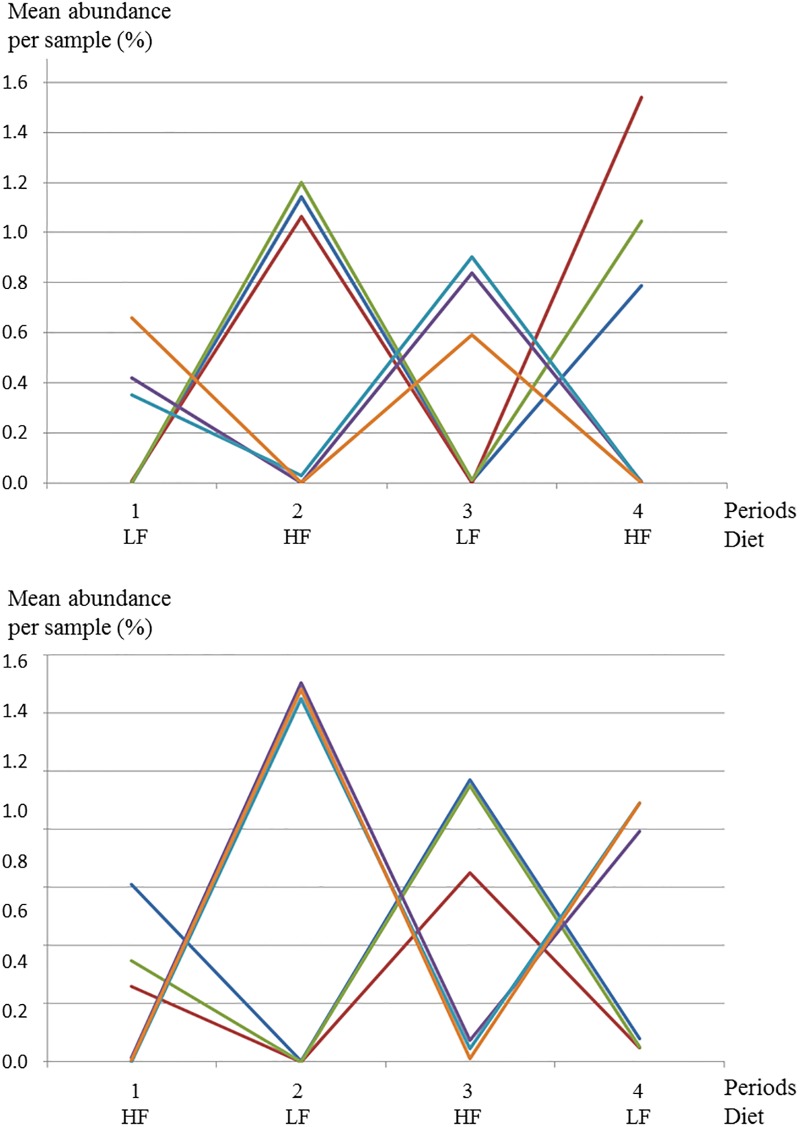
Variations of the relative abundancy of predictive OTUs of diet effect. The three most contributive OTUS from Low-fiber diet (LF) (OTU 514: purple, OTU 1140: light blue, OTU 923: orange) and High-fiber diet (HF) (OTU 792: dark blue, OTU 689: red, OTU 1940: green) are extracted from the sPLS-DA analysis using the contribution on the first principal component.

Irrespective to the breed and the period, microbiota composition was significantly influenced by dietary treatment: 1,641 out of 2,041 OTUs were differentially abundant between LF and HF diets. Unsurprisingly considering the numerous OTUs impacted, sPLS-DA discriminated the LF and HF diets with 3.9% error-rate (Fig 5) and 31 OTUs were used for the separation. The diet prediction was not altered by the previous diet or previous switches of diets showing the resilience of the biomarkers. When pigs switched from the LF to the HF diet or conversely, the abundance of the 31 OTUs used as predictors increased or decreased drastically (Fig 6). The 31 predictors have been selected among all OTUs independently of their rarity. Ranking all 2,041 OTUs according to their mean abundance, from frequent OTUs to rare ones, the 6 biomarkers abundant in the HF diet ranged from rank 3 to 1,906 and the 25 OTUs abundant in the LF diet ranged from rank 92 to 1,866. Among the 6 biomarkers abundant in the LF diet were 2 *Treponema*, 1 *Acidaminobacter* and 2 unclassified OTUs from *Bacteroidia* class. As for these 6 OTUs, the 25 biomarkers abundant in the HF diet belonged to the same phyla (12 *Bacteroidetes*, 8 *Spirochaetes* and 6 *Firmicutes*). At family level, 4 OTUs were *Prevotellaceae*, 4 *Clostridiaceae*, 1 *Clostridium* (*sensu stricto* genus), 4 *Spirochaetaceae* (1 *Treponema* and 3 unclassified genera), 1 *Peptostreptococcaceae* (*Clostridium XI* genus) and 13 were unclassified (S1 Table).

### Relationships between digestibility and microbiota

According to the influence of dietary fiber content on gut microbiota composition, correlations between OTU abundance and digestibility of energy, N and cell wall components were calculated within each dietary treatment.

**Fig 7:**
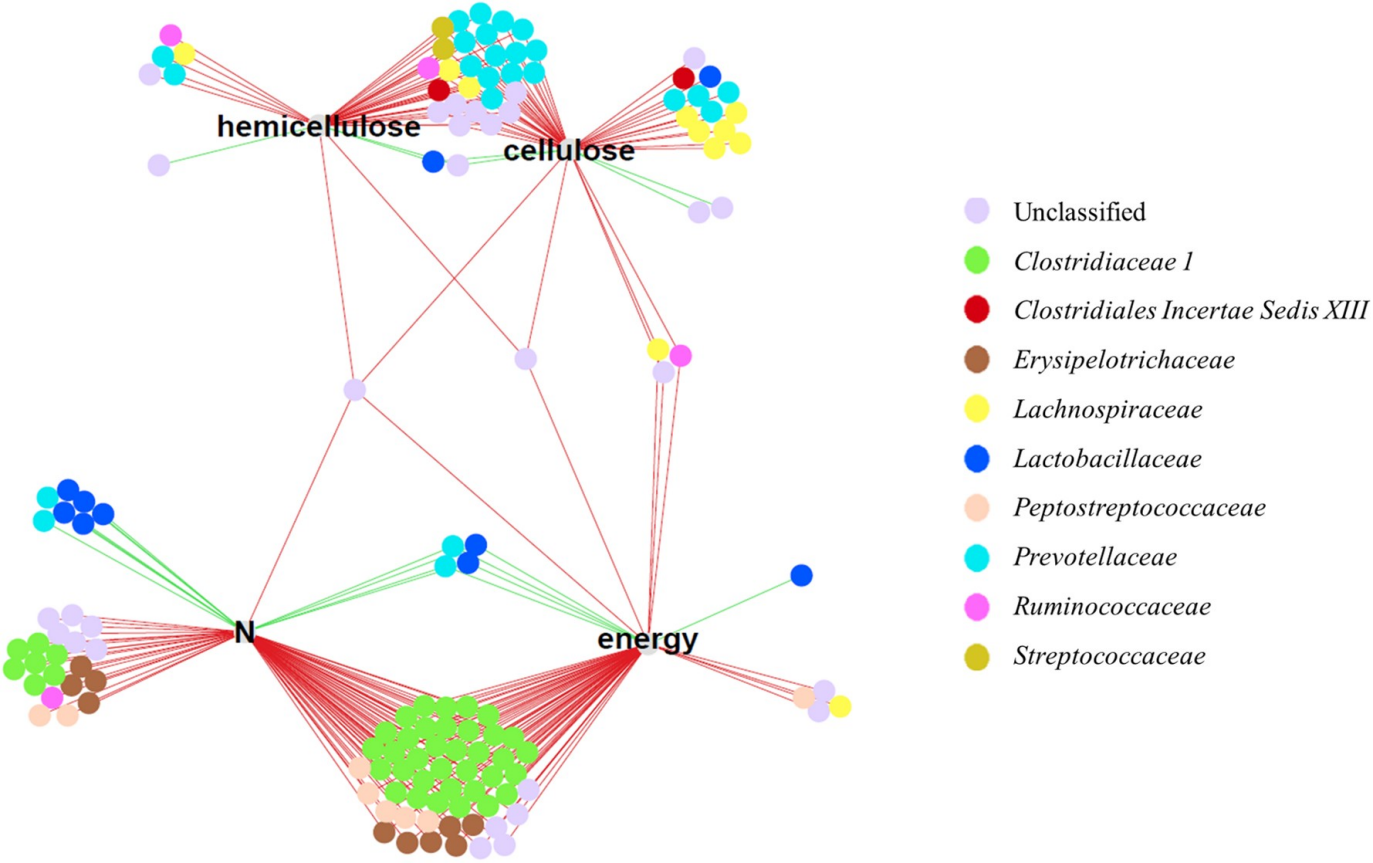
Correlated OTUs (represented by dots colored according to the taxonomical family of the corresponding bacteria) to digestibility coefficients (energy, N, hemicellulose and cellulose) in low-fiber diet through Pearson correlation. The negative and positive correlations are represented by red and green lines, respectively.

In the LF diet, 83 and 65 OTUs were significantly correlated (P < 0.05) with the digestibility coefficient of N and energy, respectively, and 49 and 37 OTUs were correlated with the digestibility coefficients of cellulose and hemicellulose, respectively (Fig 7 and S3 Table). Each OTU was either positively or negatively correlated with one or more digestibility coefficient. The significant correlation coefficients ranged from 0.29 to 0.36 in positive correlations (0.31 on average) and from -0.29 to -0.45 in negative correlations (-0.34 on average). In HF diet, no OTU were found to be significantly correlated with digestibility coefficients (S5 Fig).

*Clostridiaceae 1* (*Clostridium sensu stricto* and unclassified at genus level), *Erysipelotrichaceae* (*Turicibacter*) and *Peptostreptococcaceae* (*Clostridium XI*) were negatively correlated with energy and N digestibility coefficient whereas *Lachnospiraceae* (*Blautia*, *Coprococcus* and *Dorea* genera) were negatively correlated with the digestibility of energy, cellulose or hemicellulose. Two OTUs belonging to *Clostridiales Incertae Sedis XIII* and 4 *Streptococcus* were negatively correlated with cell wall components digestibility. *Ruminococcaceae* negatively correlated with cellulose, hemicellulose, N and energy digestibility.

*Prevotellaceae* (mainly *Prevotella* genus) were negatively correlated with the digestibility of cell wall components except for 6 unclassified *Prevotellaceae* which were positively correlated with energy and N digestibility.

*Lactobacillaceae* were positively correlated with N, energy, cellulose and hemicellulose digestibility except one negatively correlated with cellulose digestibility. The only genus represented in this family was *Lactobacillus*.

## Discussion

Most bacteria in the pig gut in the current experiment (88%) were *Firmicutes* and *Bacteroidetes* in agreement with previous studies [9, 28]. *Proteobacteria*, *Spirochaetes* and *Synergistetes* were also present in matured pig feces microbiota but in smaller amount (< 8% each), as previously shown [29]. The gut microbiota in our experiment were dominated by *Ruminococcus* and *Treponema* suggesting that the pigs belonged to the same enterotype [10] and that the analysis methods designed to identify correlations within homogenous groups were relevant.

During the first period of our experiment, the microbiota of the Large White, Duroc and Pietrain animals differed when fed the LF diet. It should be noted that the animals from each breed originate from a different farm but were housed from weaning onwards in the same conditions. Because of a possible confusion between the farm origin and the breed, no conclusion can be made regarding the impact of breed on the microbiota. However the observed differences in microbiota according to the breed/origin emphasized variability in the animals of our experiment. Then further conclusions about the relationships between microbiota and digestibility are robust with respect to the origin or the breed of the pigs.

### The increase of digestibility in aging animals was not linked to the microbiota

According to our results, the digestive utilization of energy, N, OM and cell wall components linearly increased over time. This linear increase is in agreement with published values: energy digestibility increases by 0.9 point per 10 kg BW [27]. In our study, the energy digestibility increased by 0.6, 0.8 and 1.0 point in Duroc, Large White and Pietrain pigs, respectively. These lower results compare to the previous ones on Large White growing pigs [27] might be due to the lower daily feed intake in our experiment.

Regarding the microbiota composition, the effects of aging were not as clear as the effect of diet. This might be due to the period alternation with different diets. In addition, diversity indexes and microbiota composition remain generally stable after 3 months of age in growing pigs [5]. As a consequence, the lack of change in microbiota composition with the age might reflect the stability of microbiota composition in 3 to 5 months old pigs. Since the improvement of energy, N or cell wall components digestibility over time seemed unrelated to microbial changes in the hindgut, other factors were likely to be responsible. For example the transit rate changes during the pig growth [30, 31] which could cause the digestibility improvement with time.

### Robust effect of the diet on digestibility and microbiota

In the present study, fibers decreased the energy and OM digestibility as observed previously [19]. The present study shows a 0.8% reduction of energy digestibility for each 1% increase in NDF content, which is in agreement with the 1% reduction that was previously reported in growing pigs [20].

Fibers also decreased the digestibility of N which may be due to mechanical erosion of the gut epithelium. This erosion led to more endogenic secretion mainly constituted with proteins [32] and thus increased the N losses in feces. Furthermore, a previous study [15] observed an increase in bacterial counts with high-fiber diet possibly resulting in an increase of ureic N released in the gut [33] and therefore intensifying fecal N losses and decreasing its digestibility. Because insoluble fibers as wheat bran or soya hulls kept water in their matrix [34], nutrients like proteins were trapped and became less available for enzyme activity [35]. In addition, fibers mechanically acted on gut wall through the increase of the volume to digest [36, 37] resulting in an increase of colon motility [38] and a decrease of the transit time [39]. The exceeding proteins in the digesta, their lower availability and the decrease of transit time increased the loss of N in the feces, participating to the decrease of the apparent digestibility of N as in our study.

Replacing part of the feed by ingredients containing fibers also had a strong impact on gut microbes. About 80% of the OTUs were differentially abundant between diets irrespective to the period or the breed. The impacts of dietary fiber addition on microbiota composition have been extensively described in pigs [8, 40, 41] or in humans [42–44]. The most likely mechanism is the production of SCFAs [45, 46], which have been shown to exert multiple beneficial effects on host energy metabolism and on the health in the small intestine. As an example, proliferation of the epithelial cells and cell turnover were stimulated by fermentations products [47, 48], related to an improved digestibility of cell wall components.

The microbial biomarkers used to discriminate pigs fed LF to those fed HF diets in our experiment belonged to *Bacteroidia*, *Clostridia* and *Spirochaetia* classes, *Prevotellaceae*, *Clostridiaceae* and *Spirochaetaceae* families. These results are in agreement with data published in pigs [14] or in humans [43, 49]. A stimulation of the glucose metabolism induced by the ingestion of fiber rich diet by humans led to an increased *Prevotella* abundance [50]. In addition, previous studies correlated *Clostridium sp*. to the gut motility [51, 52] which is triggered by fiber ingestion. The third family mainly represented among our predictors was *Spirochaetaceae*. *Treponema* as a member of this family present pectinolytic functions [53] suggesting that its abundance may be highly affected by modifications in the dietary fiber content.

### Resilience of the microbial biomarkers to diet perturbations

In the present study, a three-week adaptation period to a given diet was sufficient to stabilize the relative abundance of the microbial biomarkers of the diet suggesting a resilience of the corresponding bacterial populations. Similarly, it has been demonstrated that an equilibrium of the structural and functional composition of the microbiome is reached three weeks after a dietary change in pigs [54]. In addition, previous study analyzed the gut microbiota in mice fed alternately a fiber rich diet followed by a low-fiber diet. The initial microbiota abundance partially recovered in a third period when the mice were fed the first fiber rich diet again [55], showing the resilience of part of the microbiota. According to Camody et al. [56], in mice, microbial response to a diet perturbation takes only few days to reach a new stable state and most changes in the gut microbiota are reversible. The speed of response of the pig gut microbiota to a challenged diet remains to be determined, especially when the pigs have been previously exposed to the same challenged diet.

### Correlation between digestibility and microbiota

In our experimental conditions, correlations between microbiota and digestibility of N, energy, cellulose and hemicellulose were generally low and rarely significant. Indeed, only 142 OTUs out of 2,041 were significantly correlated with digestibility coefficients with a maximum absolute correlation value reaching 0.45. Caution should be used in interpreting results of correlation analysis because a significant correlation does not necessarily imply causation. These correlation values were generally smaller than those previously reported by Niu et al. [21]. This discrepancy could be explained first by differences in the method used for assessing gut microbiota composition. Niu et al. [21] calculated their correlations at the taxonomical levels phylum, class, order, family and genus using only OTUs with full bacterial taxonomy assignation whereas our calculations were realized at OTU level and used every OTU abundance, with or without taxonomy assignation. The inter-individual variability is generally much higher for OTUs than for higher taxonomic levels, possibly leading to lower correlations values at OTU level. Comparing our correlations with every OTU grouped by taxonomical levels and those from Niu et al. [21], *Treponema* genus is positively correlated to cellulose digestibility in both studies but no other correspondence has been found at higher taxonomical level.

Furthermore the contribution of microbiota in nutrient digestion might be limited in growing pigs assuming that the largest amount of energy is digested in the foregut. The energy digested in the hindgut represents only 7 to 18% depending on the dietary fiber content and composition [1]. At the same time, the biggest fraction being fermented in the hindgut (non-starch polysaccharides) by the microbiota accounts for 50 to 77% of energy disappearance for the hindgut of the pig. In addition, the relation between microbiota composition and energy harvest is disputed [57, 58]. Surprisingly however significant correlations were detected in the LF diet rather than in the HF diet in which microbial fermentation was expected to play a larger role.

In the LF diet, we reported significant correlations between microbiota composition and digestibility coefficients of N, energy, cellulose and hemicellulose and these correlations were robust because they were independent of the period, the breed and the farm origin of the animals. The correlated OTUs belonged to 9 bacterial families. Any discussion about the functional impact of microbes on digestibility is more relevant at the OTU level rather than using inferring from higher taxonomic affiliations. Indeed, among the OTUs significantly correlated with digestibility coefficients, the OTUs belonging to 7 families over 9 were either positively or negatively correlated with digestibility coefficients. For example the correlated OTUs belonging to *Clostridiaceae* were all negatively correlated with energy and N digestibility. However, *Lactobacillaceae* and *Prevotellaceae* families were represented by both positively and negatively correlated OTUs. Therefore it is not possible to conclude about the impact of a whole family on digestibility and the relationships between microbiota and digestibility should be described at OTU level.

Surprisingly, OTUs belonging to *Clostridiaceae* family were not associated with cell wall components digestibility in our study even though they have been previously positively correlated with apparent crude fiber digestibility [21] and tends to be associated to dietary fiber metabolism [59]. Intriguingly, OTUs belonging to the *Ruminococcaceae* and to the *Lachnospiraceae* families were negatively correlated with cell wall component digestibility in our study although they possess genes coding for proteins (xylanases, cellulases) to degrade a wide variety of polysaccharides [60] and may have protection properties against diseases, as their abundance are higher in healthy gut of piglets compared to sick ones [61]. Therefore their negative impact on cell wall components digestibility was not backed up by the literature.

Our study revealed that the cell wall components digestibility was negatively correlated with OTUs belonging to *Prevotella*. The fermentation abilities of *Prevotella* genus cannot be inferred because *Prevotella* (*albensis*, *brevis*, *bryantii* and *ruminicola*) poorly ferment hemicellulose [62] but *Prevotella copri* abundance was linked to an improved capacity to ferment complex polysaccharides [50]. *Prevotella* were also related to chronical inflammatory diseases, mainly studied in humans [63, 64]. But this cannot be generalized to all *Prevotellaceae* since OTUs from *Prevotellaceae* family were found in higher abundance in healthy piglets compared to diarrheic ones [61]. Therefore, some *Prevotellaceae* may have a positive impact in gut health which could explain the positive correlation between energy and N digestibility and undefined *Prevotellaceae* in our study.

As previously reported by Vigors et al. [65], our study highlighted a positive link between the abundance of OTUs belonging to *Lactobacillaceae* and digestibility of N and energy. Some bacteria belonging to *Lactobacillaceae* family seems to improve the nutrient digestibility through the fermentation. As an illustration, *Lactobacillus fermentum* supplementation was related to an improved apparent digestibility of crude protein in weaned pigs [66]. Likewise, a *Lactobacillus acidophilus* supplementation increased linearly the apparent digestibility of N, possibly through the stimulation of the hindgut fermentation [67]. Such a stimulation could be primarily due to the exopolysaccharides produced by *Lactobacillus* [68]. However, *Lactobacillaceae* are also known to improve the health of the small intestine in humans as in animals [69, 70] as a probiotic. As a consequence, the positive effect of *Lactobacillaceae* on the ability to digest feed could also be partly due to an indirect effect on gut health.

Surprisingly, no significant correlation was reported between OTU abundance and digestibility coefficients in the HF diet. According to Martens et al. [71] several bacteria have the ability to metabolize the same dietary fiber substrates. Thus, when a dietary perturbation occurs, different bacterial strains may compete for the substrate and some will expand at the expense of others [44, 72]. No similar reaction in microbiota composition can be observed among the pigs fed a challenged HF diet which prevent from seeing significant correlations between digestibility coefficients and microbiota composition in the HF diet. This assumption is purely speculative and requires further analyses. However the digestion might be improved by providing specific enzymes in the feed according to the challenged diets.

## Conclusion

In growing-finishing pigs, microbiota composition can be used to discriminate with accuracy the animals according to their dietary treatment using 31 bacterial biomarkers. Even though global fecal microbiota composition remained stable across time, our study revealed that three weeks of adaptation to a new diet are sufficient to observe the resilience of these biomarkers. Furthermore, in case of LF diet, protein and energy digestibility coefficients were positively correlated with OTUs belonging to *Lactobacillus* whereas OTUs belonging to *Clostridiaceae*, *Lachnospiraceae* and *Prevotella* were negatively correlated with nutrients and cell wall components digestibility. This suggests beneficial or detrimental properties of these bacteria in pig digestion; however their underlying biological functions remain to be determined.

## Supporting information

S1 Fig Pipeline of analyses applied on the digestibility coefficients (DC) and the operational taxonomic units (OTUs): Pearson correlation, variance analysis (ANOVA), global linear model (glm) and sparse partial least square discriminant analysis (sPLS-DA). (TIF)

S2 Fig No significant variation in microbiota composition at a phylum level between diets (LF: Low-fiber diet; HF: High-fiber diet), breeds (DU: Duroc; LW: Large-White; PI: Pietrain) and periods (per1 to per4) in Khi^2^ tests. (TIF)

S3 Fig No significant differences between diets (LF: Low-fiber diet; HF: High-fiber diet), breeds (Pietrain, LW: Large-White, Duroc) or periods (periods 1 to 4) in terms of diversity according to the observed abundance (Observed index), the Shannon or the Simpson indexes. (TIF)

S4 Fig Score plot of two-component sPLS-DA model showing feces samples clustering according to the period with percentage of variance captured for each principal component (Δ: Period 1, □: Period 2, O: Period 3, +: Period 4) for the animals fed low-fiber diet or high-fiber diet. According to the cross-validation permutation test, the misclassification error-rates are respectively 52% and 51%.
(TIF)

S5 Fig Repartition of the OTUs according to the absolute value of their correlation with N, energy, cellulose and hemicellulose digestibility coefficients (figures a, b, c and d, respectively) in high-fiber diet (HF) or in low-fiber diet (LF). The significant correlations are represented in blue.
(TIF)

S1 Table Contribution and taxonomy of the predictive OTUs of high-fiber (HF) vs low-fiber (LF) diet in sPLS-DA. (CSV)

S2 Table Contribution and taxonomy of the predictive OTUs of breed/farm in sPLS-DA in period 1 for pigs fed low-fiber diet. (CSV)

S3 Table Pearson correlations and taxonomy of the OTUs correlated with the digestibility coefficients of energy, N, cellulose and hemicellulose. (CSV)

S4 Table Phenotypic dataset. (CSV)
