# Supplementary figures and images for: Effect of dietary fiber content on nutrient digestibility and fecal microbiota composition in growing-finishing pigs
Source: PLoS One. 2018 Oct 24;13(10):e0206159. doi: 10.1371/journal.pone.0206159 (PMC6200266; doi:10.1371/journal.pone.0206159)

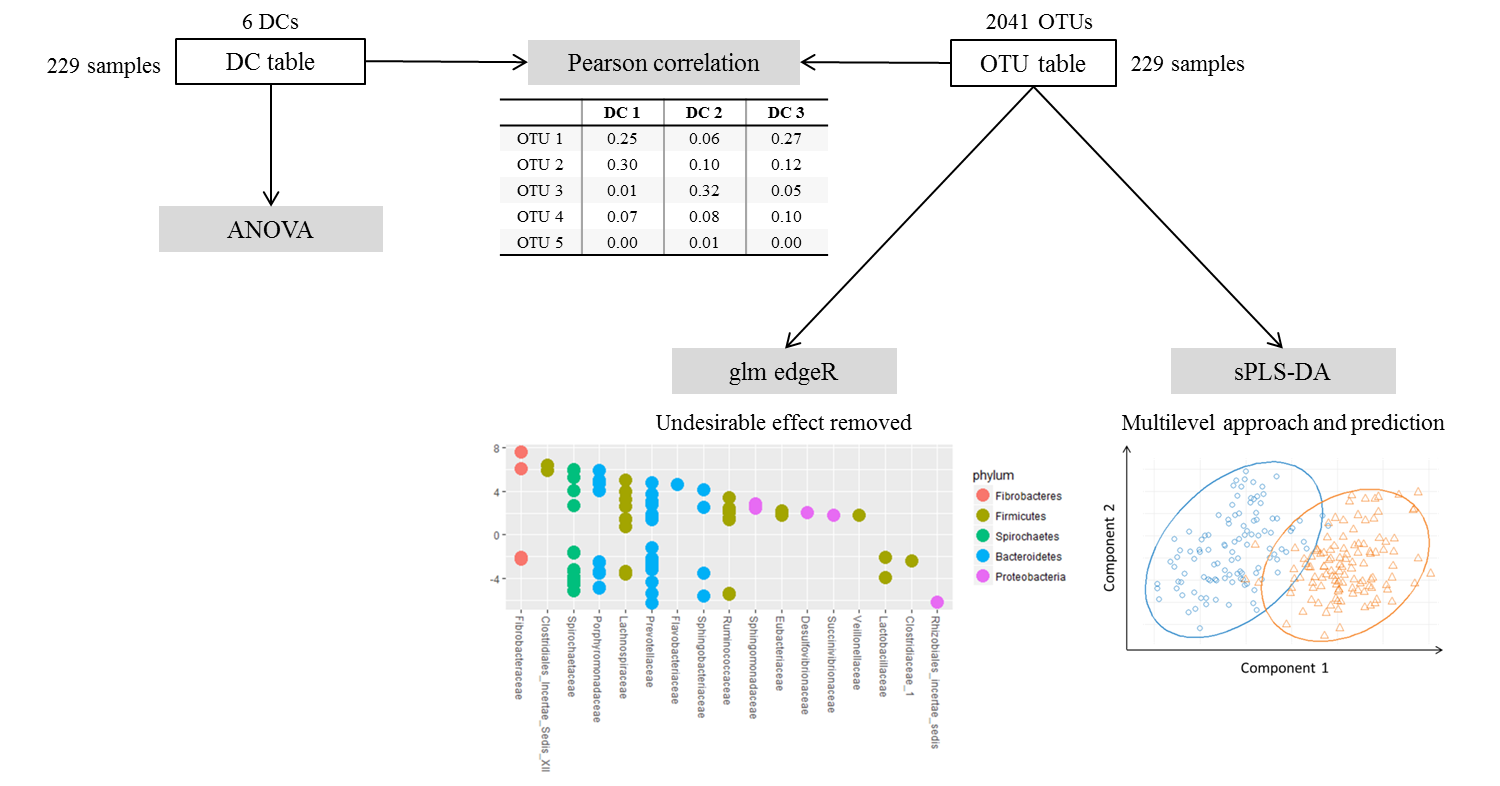

Supplement: S1 Fig — (TIF) [file pone.0206159.s001.tif]

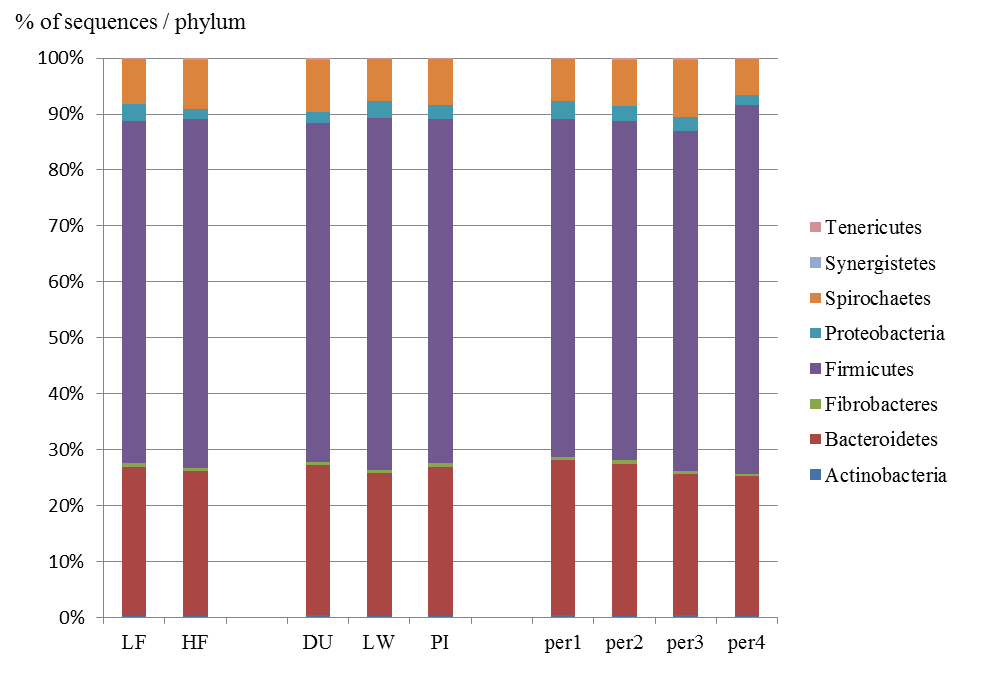

Supplement: S2 Fig — (TIF) [file pone.0206159.s002.tif]

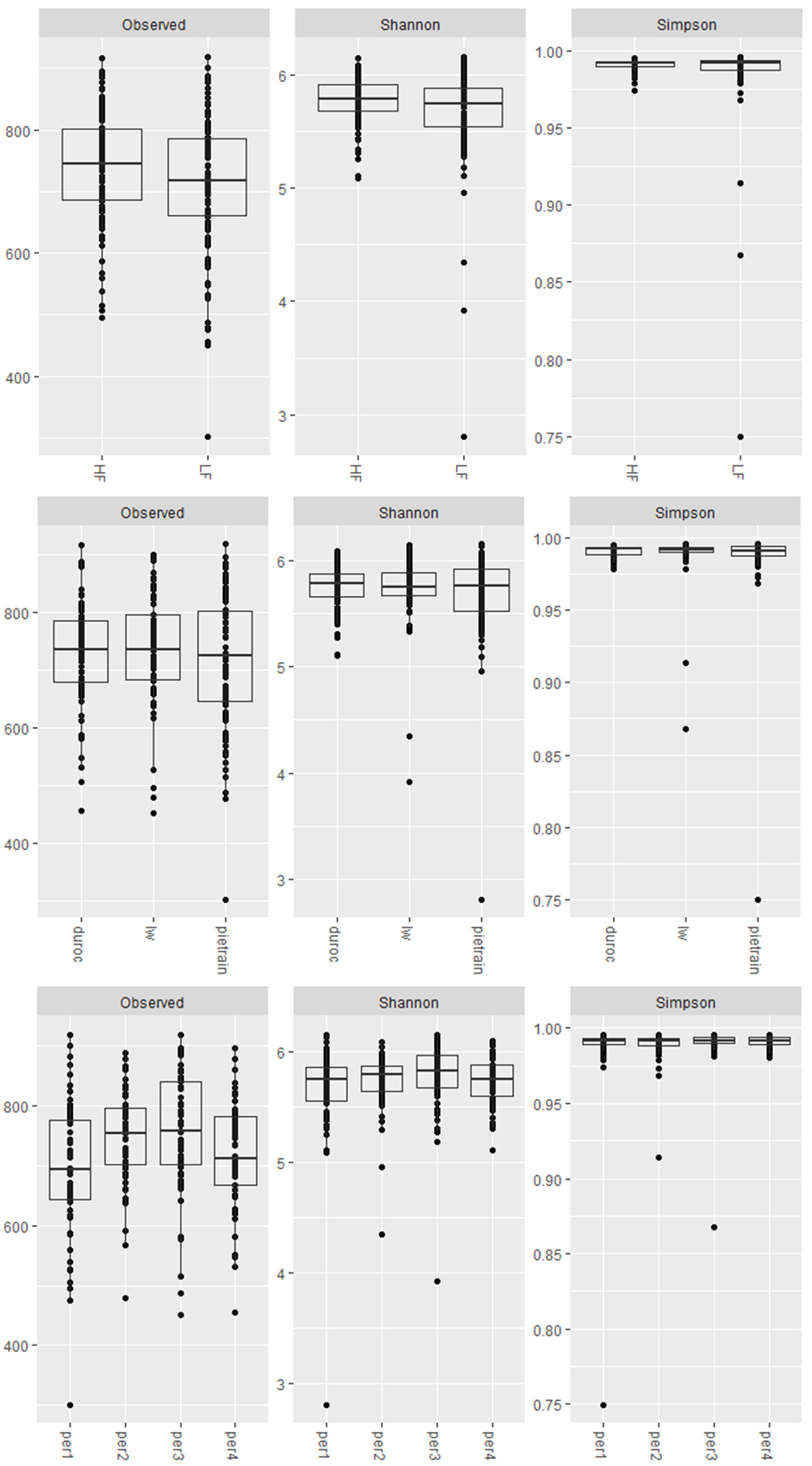

Supplement: S3 Fig — (TIF) [file pone.0206159.s003.tif]

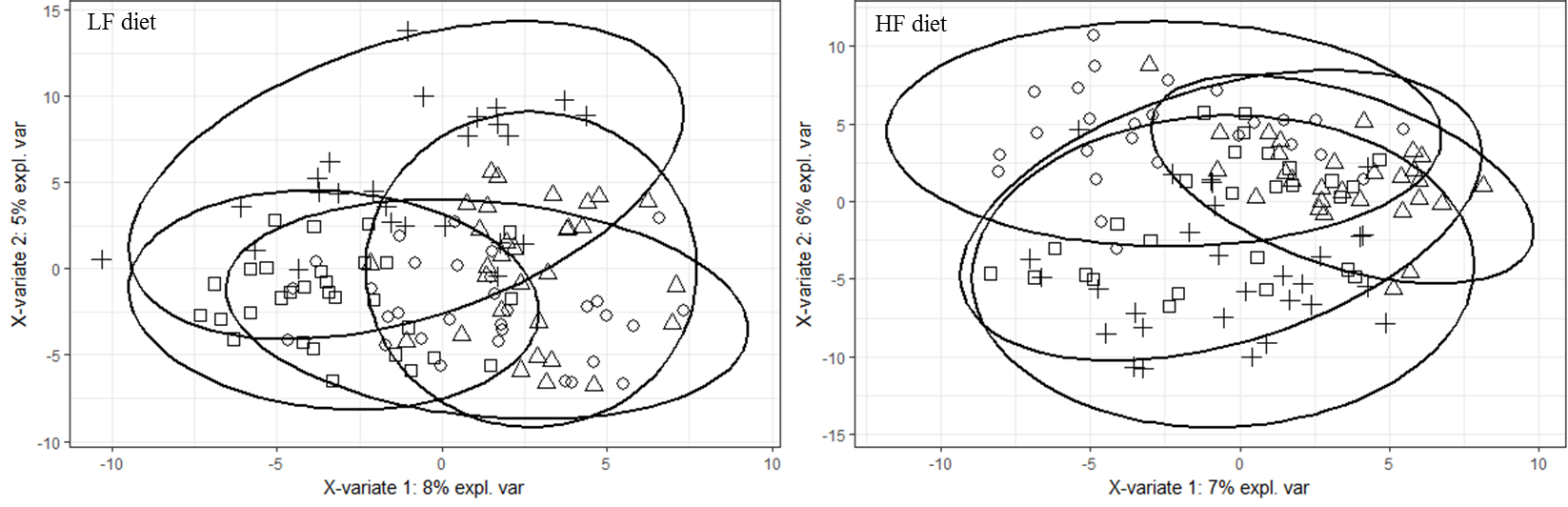

Supplement: S4 Fig — According to the cross-validation permutation test, the misclassification error-rates are respectively 52% and 51%. (TIF) [file pone.0206159.s004.tif]

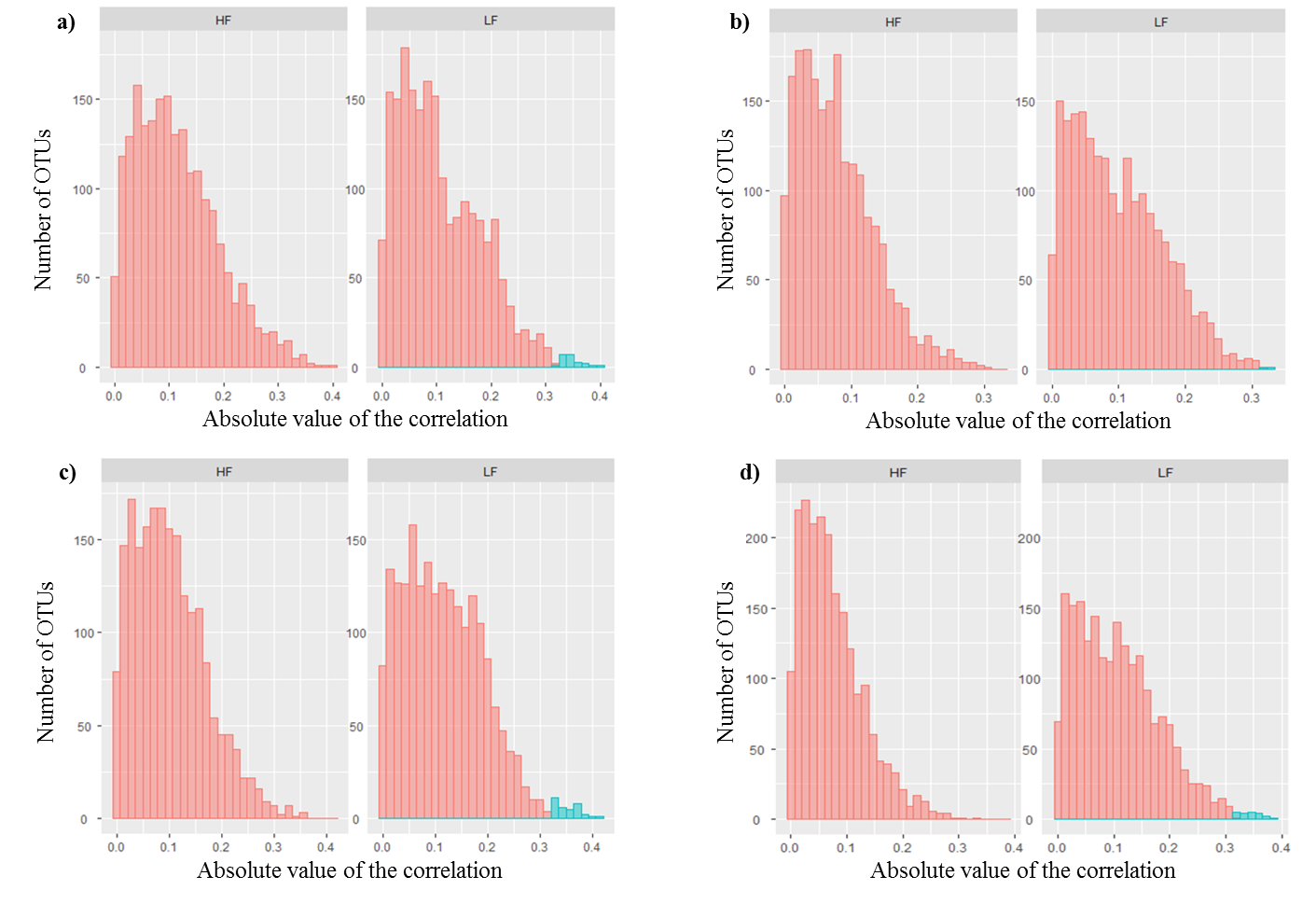

Supplement: S5 Fig — The significant correlations are represented in blue. (TIF) [file pone.0206159.s005.tif]
